# Facile One-Pot Synthesis of Nickel Nanoparticles by Hydrothermal Method

**DOI:** 10.3390/ma16010076

**Published:** 2022-12-21

**Authors:** Gawon Kim, Chan Woong Na, Yoon Myung

**Affiliations:** 1 Department of Nanoscience and Engineering, Inje University, 197 Inje ro, Gimhae 50834, Republic of Korea; 2 Dongnam Regional Division, Korea Institute of Industrial Technology, Busan 46938, Republic of Korea

**Keywords:** nickel nanoparticle, one-pot synthesis, borane-ammonia complex reduction, morphology control, hydrothermal

## Abstract

The one-pot synthesis process has emerged as an economical synthesis method without the involvement of purification or formation of intermediate compounds. Therefore, nickel nanoparticles were selectively synthesized by a simple hydrothermal method using nickel(II) chloride hexahydrate and borane–ammonia complex as a precursor and reducing agent, respectively. The morphology and crystal growth were observed by controlling the precursor concentration ratio of Ni:AB from 1:0.1 to 1:4 under various temperatures ranging from 80 to 140 degrees. In addition, we observed that the crystal growth rate under the influence of NaCl and KCl resulted in spherical Ni particles with size distributions controlled in the range of 297.65 nm to 1082.15 nm and 358.6 nm to 605 nm, respectively.

## 1. Introduction

Recently, interest in the development of catalysts capable of producing hydrogen, which is a representative alternative energy, is increasing due to the increase in the price of natural resources such as natural gas and oil [1,2,3,4,5,6,7]. Nickel is a transition metal with a face-centered cubic structure and is widely used industrially as a material for organic chemical synthesis, multilayer ceramic capacitors, steam methane reforming, dry reforming, and fuel cells, and is an important material throughout chemical and environmental industries [8,9,10,11,12,13,14,15,16,17,18,19,20,21].

Methods applied to chemical synthesis (mentioned above) use techniques such as ball milling, sol-gel, immersion, spray drying, solvothermal, and hydrothermal methods, respectively [11,22,23,24,25,26,27]. However, in the case of wet synthesis, after forming the primary intermediate compound with a reducing agent such as NaOH, KOH, ammonia, or hydrazine, an additional reduction process of two or more steps or a thermal annealing treatment in a hydrogen atmosphere after the formation of the dry powder is required [24,26,27]. In addition, since there are difficulties in fine particle refinement with impurity control associated with these two or more synthesis steps, it is necessary to develop a synthesis method that can overcome them. Recently, borane–ammonia complex (AB) has mainly been used as a hydrogen storage medium, and has attracted attention as a reducing agent capable of synthesizing various metal nanoparticles such as Au, Ag, Cu, Pd, and Ir [28,29,30]. In this study, a process that can be synthesized in one pot without an additional reduction or heat treatment was studied using the hydrothermal synthesis method using nickel(II) chloride hexahydrate as a precursor and a borane–ammonia complex reducing agent with NaCl and KCl as effective additives to the growth of spherical Ni nanoparticles. 

## 2. Experimental

All Ni nanoparticle powders were synthesized using hydrothermal synthesis. An amount of 0.35 M (4.160 g) of nickel(II) chloride hexahydrate (molecular weight (Mw): 237.69 g/mol, Aldrich, Reagent Plus grade) and a borane–ammonia complex precursor (Mw: 30.865 g/mol, Aldrich, 97%) were dissolved in D.I. water with in a molar ratio of 1:0.1 to 1:4, respectively (0.035 M (0.054 g), 0.07 M (0.108 g), 0.35 M (0.540 g), 0.7 M (1.080 g), 1.4 M (2.160 g)). These solutions were stirred for 30 min and then poured into a Teflon-lined autoclave and heated at 80~140 °C for 12 h. Synthesized Ni nanoparticle powders were washed with D.I. water and ethanol using a centrifuge and dried in an oven at 60 °C for 12 h. To observe the particle growth effect of the nanoparticle powder according to the additive, NaCl (Mw: 58.44 g/mol, Aldrich, ACS Reagent Grade) or KCl (Mw: 74.551 g/mol, Aldrich, ACS Reagent Grade) solution was added to the nickel(II) chloride hexahydrate precursor in a molar ratio of 1:0.1 to 1:10 (0.035 M (0.130 g), 0.175 M (0.652 g), 0.35 M (1.305 g), 1.75 M (6.523 g), 3.5 M (13.046 g)).

The shape and particle size of the nanoparticle powders were measured using a field emission scanning electron microscope (FE-SEM, Hitachi, Su-5000) and field emission transmission electron microscope (FE-TEM, FEI Technai G2 200 kV). The crystal structure of the nanoparticle powder was analyzed with a powder X-ray diffractometer (XRD, X-ray Diffractometer system, Rigaku, Miniflex 600) using a Cu-Kα radiation light source (λ = 0.15056 nm). The chemical composition change and surface bonding state of the nanoparticles were determined using X-ray photoelectron spectroscopy (XPS, X-ray photoelectron Spectroscopy, Thermo Scientific, Sigma Probe) with a photon energy of 1486.6 eV (Al Kα). 

## 3. Results and Discussions

**Figure 1:**
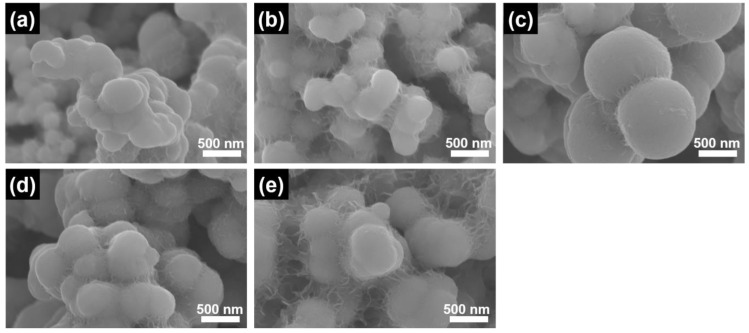
SEM images of Ni nanoparticles synthesized at 80 °C with different concentration ratios of Ni:AB: (**a**) 1:0.1, (**b**) 1:0.2, (**c**) 1:1, (**d**) 1:2, and (**e**) 1:4, respectively.

Figure 1 shows the SEM images of Ni nanoparticle powder synthesized at 80 °C for 12 h using nickel(II) chloride hexahydrate and AB as precursors.

**Scheme 1:**
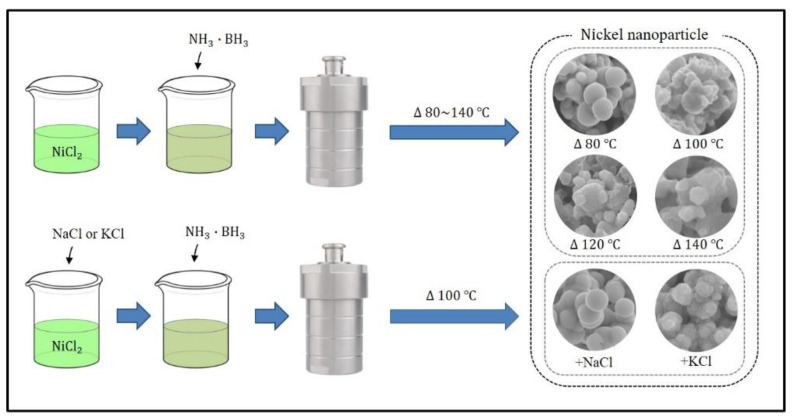
Scheme of synthetic steps of one-pot-synthesized Ni nanoparticles.

Scheme 1 shows that the size and shape of Ni particles change depending on the temperature and the presence or absence of additives when the Ni:AB value is 1:1. In addition, we experimented with different concentrations for each temperature. Figure 1 shows SEM images of Ni particles synthesized for 12 h at 80 °C When the Ni:AB precursor concentrations were 1:0.1, 1:0.5, 1:1, 1:2, and 1:4, the average size of the aggregated particles was determined in Figure 1a as ~300 nm, in (b), it was observed to be ~200 nm, (c) ~500 nm, (d) ~300 nm, (e) ~250 nm, respectively. As the concentration of AB increases, Ni in the form of an agglomerated amorphous film is observed (Figure 1e). EDX data shows that there are no impurity components without Ni or O (Figure S1). 

**Figure 2:**
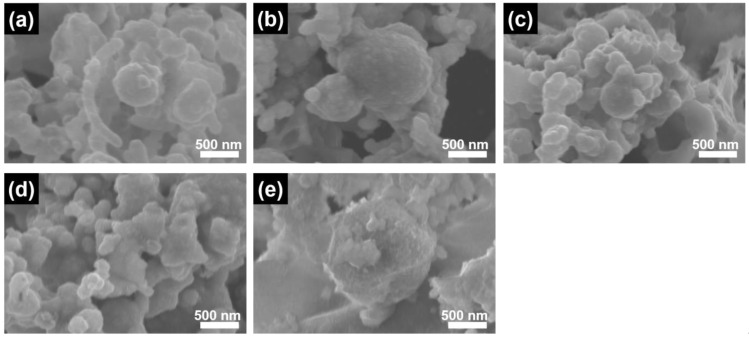
SEM images of Ni nanoparticles synthesized at 100 °C with different concentration ratios of Ni:AB: (**a**) 1:0.1, (**b**) 1:0.2, (**c**) 1:1, (**d**) 1:2, and (**e**) 1:4, respectively.

The SEM images of samples synthesized at 100 °C are shown in Figure 2. When the Ni:AB precursor concentration increases from 1:0.1 to 1:4 at 100 °C, it can be seen that the proportion of aggregated particles increases compared to Figure 1. For samples subjected to a reaction temperature of 100 °C, a film-like amorphous phase was not observed, which is the result of increasing solvation activity near the melting point of AB (m.p. 104 °C). [31] EDX data show there are no impurity components without Ni or O (Figure S2). 

**Figure 3:**
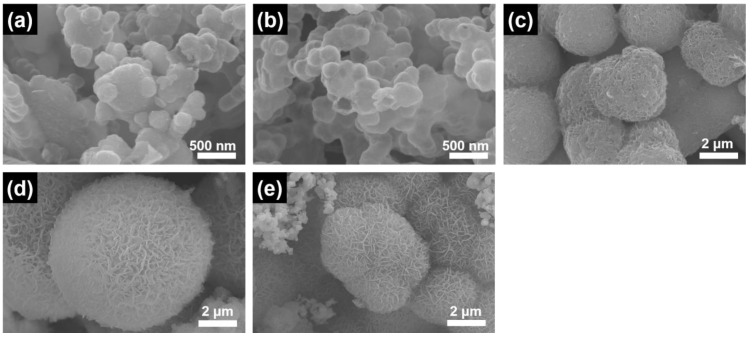
SEM images of Ni nanoparticles synthesized at 120 °C with different concentration ratios of Ni:AB: (**a**), 1:0.1, (**b**) 1:0.2, (**c**) 1:1, (**d**) 1:2, and (**e**) 1:4, respectively.

Figure 3 shows SEM images of Ni particles synthesized for 12 h at 120 °C with Ni:AB precursor ratios of 1:0.1, 1:0.5, 1:1, 1:2, and 1:4, respectively. Figure 3a shows that when the ratio is 1:0.1, the particle size is agglomerated, with a distribution of 100 nm to 400 nm, and Figure 3b shows that the mulberry-like particles were distributed with an average size of ~200 nm. Figure 3c–e show that when the Ni:AB precursor ratios are 1:1, 1:2, and 1:4, the average particle size is ~3 μm, ~5 μm, and ~2.5 μm, respectively. Since the decomposition of AB proceeds at temperatures around 110 °C, it is considered that as the concentration of AB increases, the reducing power of the surface decreases due to dehydrogenation [32]. EDX data show there are no impurity components without Ni or O (Figure S3). We performed FE-TEM measurements on a representative 1:0.2 sample. The low-magnification TEM image shows individual Ni nanoparticles with an average size of 200 nm (Figure S4a). Figure S4b shows the lattice-resolved image of Ni nanoparticles. A d-spacing of 2.0Å is observed, which is the (111) plane of a face-centered cubic Ni structure (JCPDS No. 87-0712, a = 0.3523 nm).

**Figure 4:**
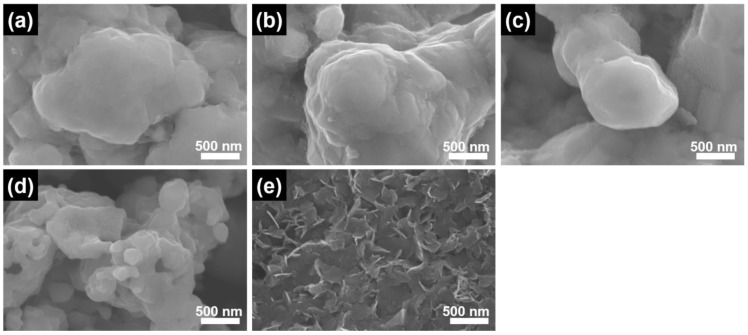
SEM images of Ni nanoparticles synthesized at 140 °C with different concentration ratio of Ni:AB as (**a**), 1:0.1. (**b**), 1:0.2 (**c**) 1:1, (**d**) 1:2, and (**e**) 1:4 respectively.

Figure 4 shows an SEM image of Ni particle powder obtained at 140 °C. For all Ni:AB ratios, spherical particles were not observed. It was confirmed that the crystals grew in the form of agglomerated two-dimensional sheets, having a thickness of 50 nm, which was observed when the concentration of the AB precursor was 1:4. EDX data show there are no impurity components without Ni or O (Figure S5).

**Figure 5:**
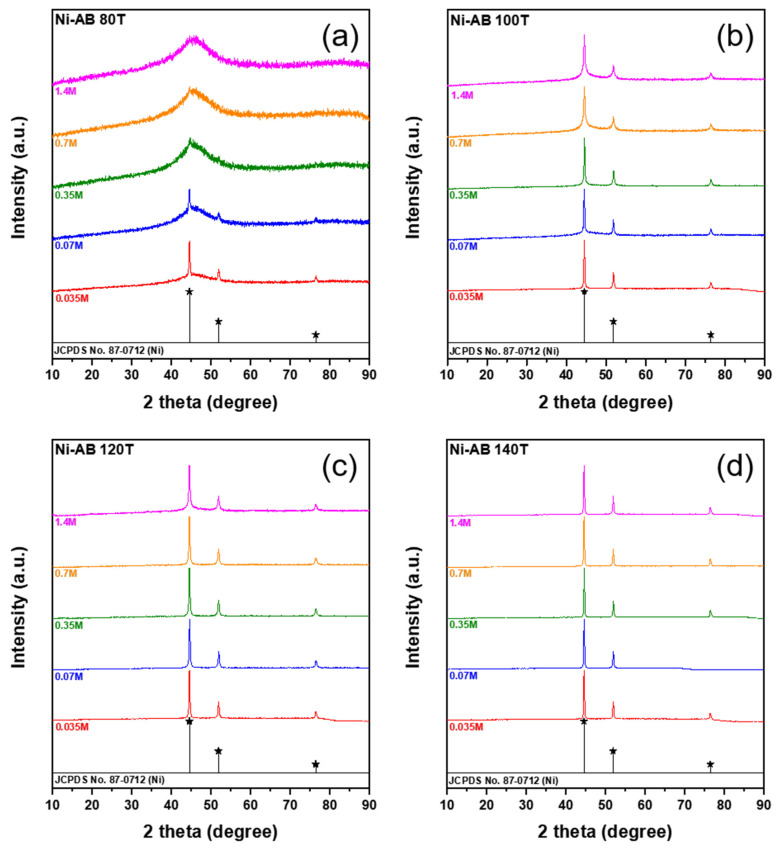
XRD patterns of Ni nanoparticles synthesized at synthesized at 0.035 M, 0.07 M, 0.35 M, 0.7 M, 1.4 M of borane-ammonia complex concentration under (**a**) 80 °C, (**b**) 100 °C, (**c**) 120 °C, and (**d**) 140 °C. (★= (111), (200), and (220) plane of face centered cubic Ni phase, respectively).

Figure 5 shows the XRD results of the synthesized Ni nanoparticle powder. The XRD pattern reveals peaks at 44.5°, 51.9°, and 76.4° over the entire temperature range. This was consistent with the face-centered cubic Ni phase (JCPDS No. 87-0712, a = 0.3523 nm) and no other impurity peaks were observed. In particular, the 80 °C sample showed a dominant peak at 44.5° of the Ni (111) at a Ni:AB concentration range of 1:1~1:4 and other peaks were weakly observed. It is believed that crystal growth is limited due to the activation of the AB complex [31].

**Figure 6:**
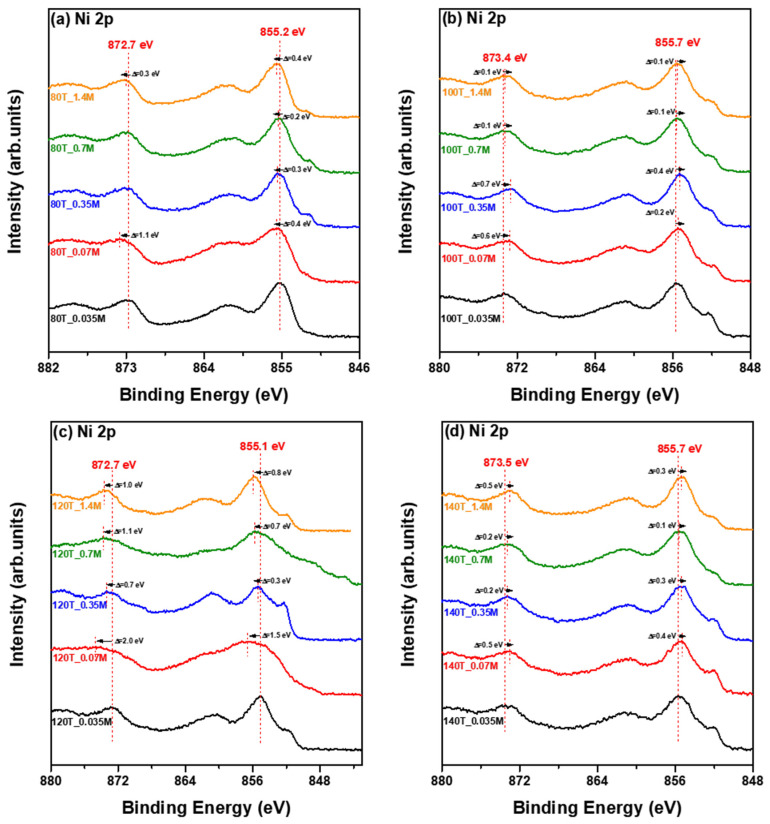
XPS Ni 2p spectra of Ni nanoparticles synthesized at 0.035 M, 0.07 M, 0.35 M, 0.7 M, 1.4 M of borane-ammonia complex concentration under (**a**) 80 °C, (**b**) 100 °C, (**c**) 120 °C, and (**d**) 140 °C, respectively.

Figure 6 shows the XPS fine spectra of Ni, confirming the surface chemical bonding state of nanoparticle powders synthesized at various temperatures and concentrations. For the 80 °C sample, the main peaks of Ni 2p_3/2_ and Ni 2p_1/2_ were observed at 855.2 eV and 872.2 eV, respectively, which means that the state of the oxidation number considered as Ni(OH)_2_ exists on the surface [33,34,35] (Figure 6a). In addition, as the AB concentration increased, red shifts were observed from 0.2 eV to 0.4 eV. It was confirmed that the Ni^0^ state existed at 852.6 eV when the Ni:AB precursor concentration ratio was 1:1 and 1:2. In Figure 6b, it is shown that 100 °C samples reveal peaks of Ni 2p_3/2_ and Ni 2p_1/2_ at 855.7 eV and 873.4 eV, respectively. The entire range of the Ni:AB precursor concentration ratio from 1:0.1 to 1:4 contains the Ni^0^ state at 852.4 eV. In the case of the 120 °C samples, in Figure 6c, the main peaks of Ni 2p_3/2_ and Ni 2p_1/2_ were observed at 855.1 eV and 872.7 eV, respectively. In particular, when the ratio of the Ni:AB precursor is 1:2, it can be seen that the full width at half maximum is the largest. It was considered that the proportion of exposed Ni(OH)_2_ or NiOOH increases as the surface area increases, as shown in Figure 3. Regarding the 140 °C samples in Figure 6d, the entire main peaks of Ni 2p_3/2_ and Ni 2p_1/2_ were observed at 855.7 eV and 873.5 eV, respectively, and distinct Ni^0^ peaks at 851.9 eV were observed.

**Figure 7:**
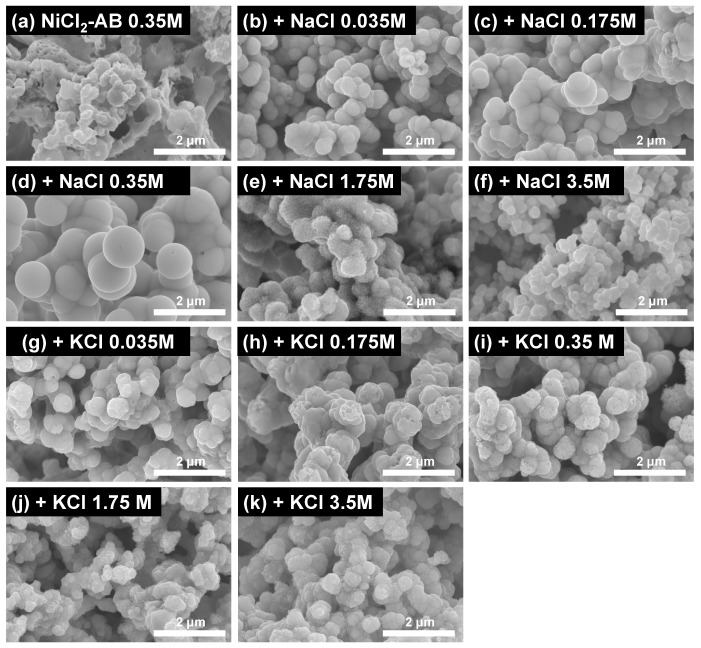
SEM images of Cl ion-controlled Ni nanoparticles: (**a**) as synthesized Ni nanoparticles without additive under 1:1 ratio of NiCl_2_ and ammonia borane complex at 100 °C, (**b**–**f**) using borane-ammonia complex with NaCl and (**g**–**k**) KCl at 1:0.1, 1:0.5, 1:1. 1:5 and 1:10, respectively.

**Figure 8:**
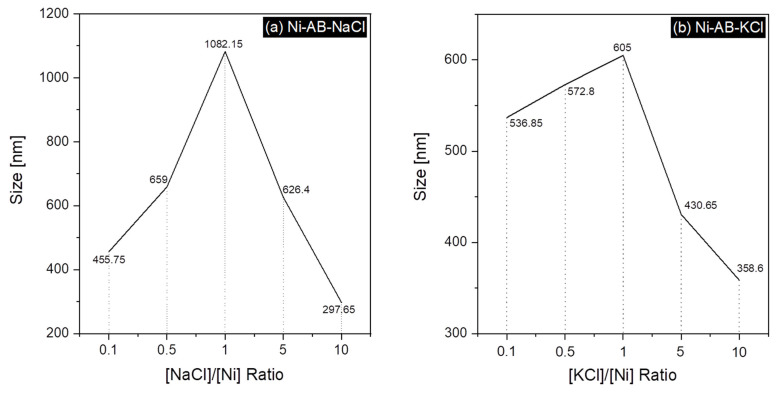
Particle size distribution of Ni nanoparticles with different concentrations of (**a**) NaCl or (**b**) KCl as an additive, respectively.

Figure 7 shows SEM images of particle growth rate changes with the addition of NaCl and KCl. The formation of spherical Ni nanoparticles was confirmed when the Ni:NaCl precursor was added in a ratio of 1:1. The average particle size distribution was 455.75 nm, 659 nm, 1082.15 nm, 626.4 nm, and 297.65 nm when the Ni:NaCl ratio was 1:0.1, 1:0.5, 1:1, 1:5, and 1:10, respectively, as shown in Figure 8a. The crystal growth reaction in response to the addition of extra Na and Cl ion concentrations was confirmed.

In the case of KCl addition, when the ratio of Ni:KCl precursor was increased to 1:0.1, 1:0.5, 1:1, 1:5, and 1:10, the average particle size was measured to 536.85 nm, 572.8 nm, 605 nm, 430.65 nm, and 358.6 nm, respectively. The overall particle size is smaller than when NaCl additive is used. It is considered that not only the Cl ion radius but also the K ion radius (r = 152 pm) are larger than that of Na ions (r = 116 pm), which was matched well, with crystal growth more effectively suppressed [36,37,38,39].

## 4. Conclusions

We studied one-pot synthesis of Ni nanoparticles using a nickel(II) chloride hexahydrate precursor and a borane–ammonia complex (AB) reducing agent using a hydrothermal synthesis method. The ratio of Ni and AB was adjusted to 1:0.1~1:4, and as a result of synthesis for 12 h at a temperature of 80 °C to 140 °C, it was confirmed that single-crystal Ni particles were formed by XRD. In addition, it was confirmed that the size distributions of spherical Ni particles were controlled in the ranges of 297.65 nm to 1082.15 nm and 358.6 nm to 605 nm by NaCl and KCl additives, respectively. We suggest that this eco-friendly one-pot synthesis method be applied to control the particle size and grain size of other transition metal nanoparticle powders to improve their chemical properties.

## Data Availability

Not applicable.

## Supplementary Materials

The following supporting information can be downloaded at: https://www.mdpi.com/article/10.3390/ma16010076/s1, Figure S1: EDX data of synthesized Ni nanoparticles at 80 °C with different concentration ratio of Ni:AB as (**a**), 1:0.1. (**b**), 1:0.2 (c) 1:1, (**d**) 1:2, and (**e**) 1:4 respectively. Figure S2. EDX data of synthesized Ni nanoparticles at 100 °C with different concentration ratio of Ni:AB as (**a**), 1:0.1. (**b**), 1:0.2 (**c**) 1:1, (**d**) 1:2, and (**e**) 1:4 respectively. Figure S3. EDX data of synthesized Ni nanoparticles at 120 °C with different concentration ratio of Ni:AB as (**a**), 1:0.1. (**b**), 1:0.2 (**c**) 1:1, (**d**) 1:2, and (**e**) 1:4 respectively. Figure S4. (**a**) TEM image of synthesized Ni nanoparticles at 120 °C with 1:0.2 ratio, (**b**) lattice resolved image of Ni nanoparticles. Figure S5. EDX data of synthesized Ni nanoparticles at 80 °C with different concentration ratio of Ni:AB as (**a**), 1:0.1. (**b**), 1:0.2 (**c**) 1:1, (**d**) 1:2, and (**e**) 1:4 respectively.
