# Supplementary material for: Facile One-Pot Synthesis of Nickel Nanoparticles by Hydrothermal Method
Source: Materials (Basel). 2022 Dec 21;16(1):76. doi: 10.3390/ma16010076 (PMC9821542; doi:10.3390/ma16010076)
Supplement: Supplementary file 1 [file materials-16-00076-s001.zip › materials-2060621-supplementary.pdf]

Supplementary Materials

Facile One-Pot Synthesis of Nickel Nanoparticles by Hydrothermal Method

Gawon Kim [1,2], Chan Woong Na [2] and Yoon Myung [2,*]

[1] Department of Nanoscience and Engineering, Inje University, 197 Inje ro, Gimhae 50834, Republic of Korea
[2] Dongnam Regional Division, Korea Institute of Industrial Technology, Busan 46938, Republic of Korea
* Correspondence: myungyoon@kitech.re.kr

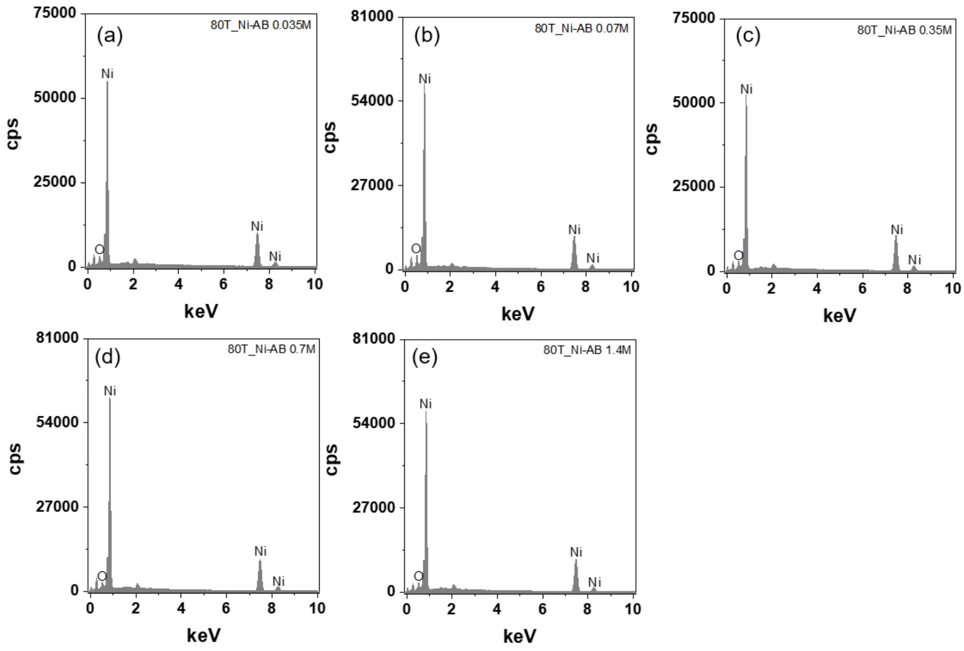

Figure S1. EDX data of synthesized Ni nanoparticles at 80 °C with different concentration ratio of Ni:AB as (**a**), 1:0.1. (**b**), 1:0.2 (c) 1:1, (**d**) 1:2, and (**e**) 1:4 respectively.

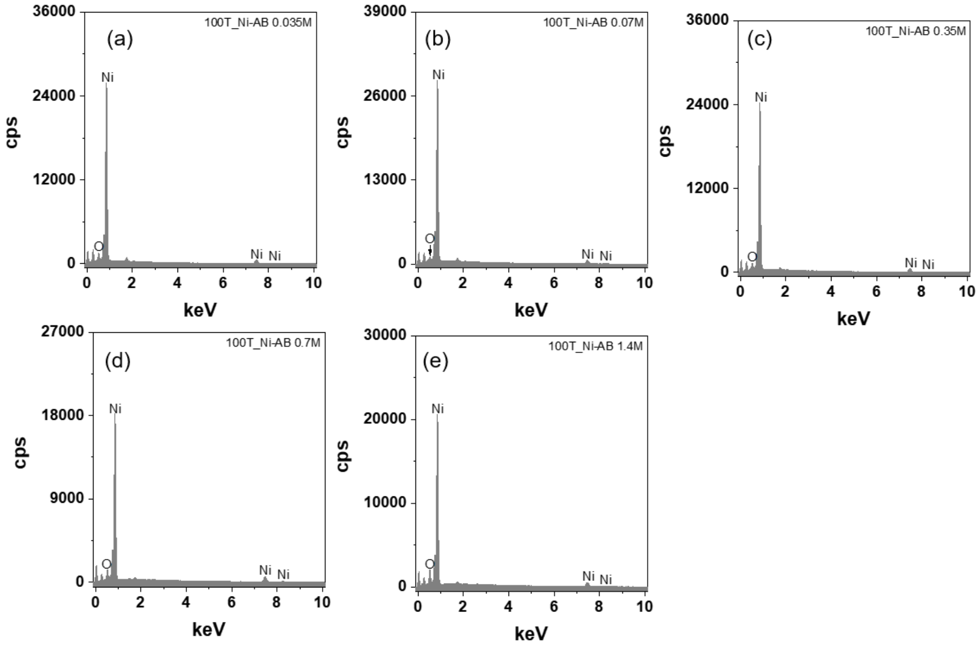

Figure S2. EDX data of synthesized Ni nanoparticles at 100 °C with different concentration ratio of Ni:AB as (**a**), 1:0.1. (**b**), 1:0.2 (**c**) 1:1, (**d**) 1:2, and (**e**) 1:4 respectively.

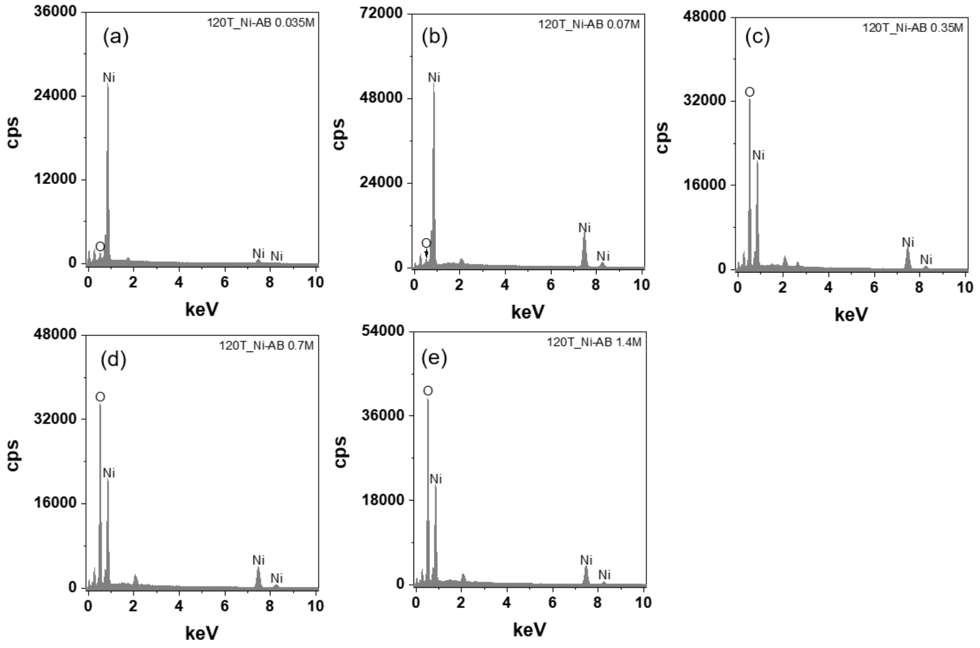

Figure S3. EDX data of synthesized Ni nanoparticles at 120 °C with different concentration ratio of Ni:AB as (**a**), 1:0.1. (**b**), 1:0.2 (**c**) 1:1, (**d**) 1:2, and (**e**) 1:4 respectively.

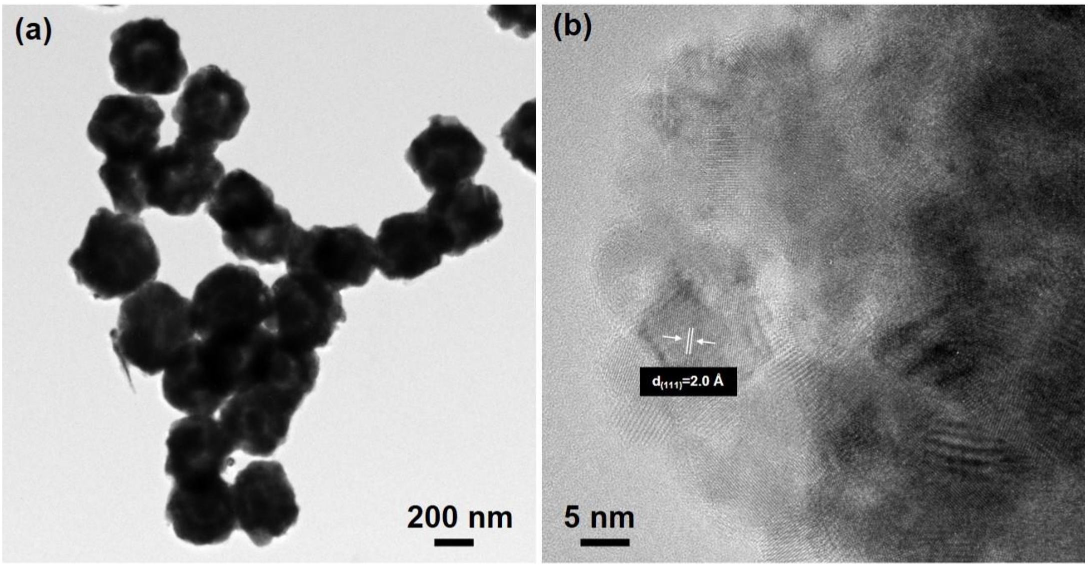

Figure S4. (**a**) TEM image of synthesized Ni nanoparticles at 120 °C with 1:0.2 ratio, (**b**) lattice resolved image of Ni nanoparticles.

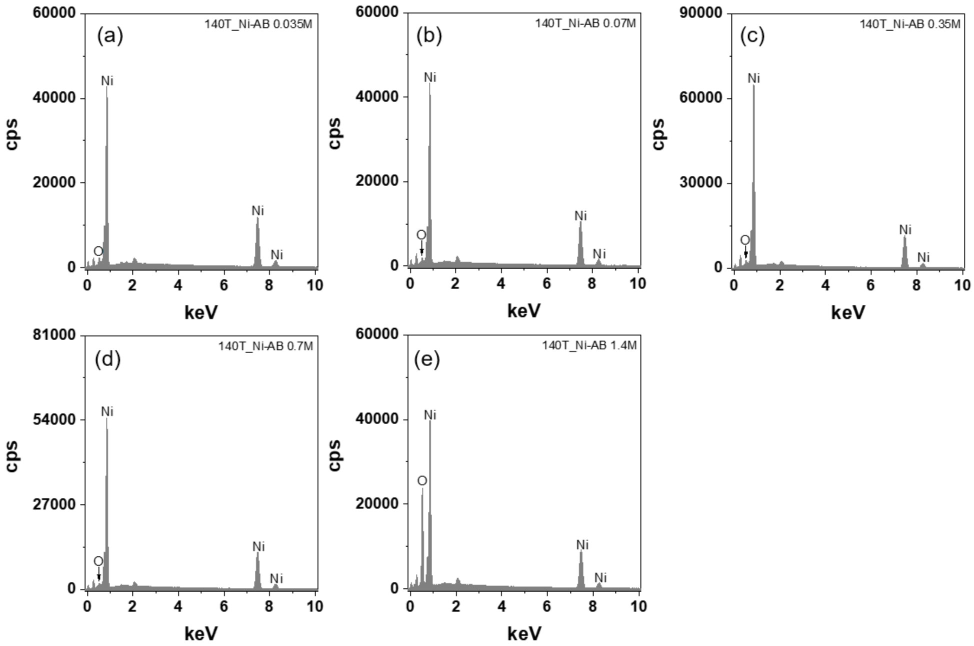

Figure S5. EDX data of synthesized Ni nanoparticles at 80 °C with different concentration ratio of Ni:AB as (**a**), 1:0.1. (**b**), 1:0.2 (**c**) 1:1, (**d**) 1:2, and (**e**) 1:4 respectively.
